# The association between vitamin D deficiency and risk of renal event: Results from the Korean cohort study for outcomes in patients with chronic kidney disease (KNOW-CKD)

**DOI:** 10.3389/fmed.2023.1017459

**Published:** 2023-02-16

**Authors:** Juyeon Lee, Eun Hui Bae, Soo Wan Kim, Wookyung Chung, Yeong Hoon Kim, Yun Kyu Oh, Yong-Soo Kim, Kook-Hwan Oh, Sue K. Park

**Affiliations:** ^1^Department of Preventive Medicine, College of Medicine, Seoul National University, Seoul, Republic of Korea; ^2^Department of Biomedical Science, College of Medicine, Seoul National University, Seoul, Republic of Korea; ^3^Department Cancer Institution, Seoul National University, Seoul, Republic of Korea; ^4^Department of Internal Medicine, Chonnam National University Medical School and Chonnam National University Hospital, Gwangju, Republic of Korea; ^5^Department of Internal Medicine, Gachon University, Gil Hospital, Incheon, Republic of Korea; ^6^Department of Internal Medicine, Inje University, Busan Paik Hospital, Busan, Republic of Korea; ^7^Department of Internal Medicine, Seoul Metropolitan Government-Seoul National University Boramae Medical Center, Seoul, Republic of Korea; ^8^Department of Internal Medicine, College of Medicine, Catholic University of Korea, Seoul, Republic of Korea; ^9^Division of Nephrology, Department of Internal Medicine, Seoul National University Hospital, Seoul, Republic of Korea; ^10^Interdisciplinary Program in Cancer Biology, College of Medicine, Seoul National University, Seoul, Republic of Korea

**Keywords:** vitamin D deficiency (VDD), renal event, CKD stage, propensity score match, cohort study [or longitudinal study]

## Abstract

**Backgrounds:**

Some observational studies have suggested a possible association between vitamin D deficiency and CKD. However, in most studies, the causality between low levels of vitamin D and risk of renal events could not be explained. We investigated the relationship between vitamin D deficiency and risk of severe CKD stage and renal event in a large-scale prospective cohort study.

**Methods:**

We used data from a prospective cohort of 2,144 patients with available information on serum 25-hydroxyvitamin D (25(OH)D) levels at baseline from KNOW-CKD, 2011-2015 were included. Vitamin D deficiency was defined as serum 25(OH)D levels < 15 ng/mL. We performed a cross-sectional analysis to elucidate the relationship between 25(OH)D and CKD stage using baseline CKD patient data. We further examined a cohort analysis to clarify the association between 25(OH)D and risk of renal event. Renal event was a composite of the first occurrence of a 50% decline in eGFR from the baseline value or the onset of CKD stage 5 (initiation of dialysis or kidney transplantation) across the follow-up period. We also investigated the associations of vitamin D deficiency with risk of renal event according to diabetes and overweight status.

**Results:**

Vitamin D deficiency were significantly associated with an increased risk of severe CKD stage – 1.30-fold (95% CI: 1.10-1.69) for 25(OH)D. Deficiency of 25(OH)D with 1.64-fold (95% CI: 1.32-2.65) was related to renal event compared with the reference. Furthermore, vitamin D deficiency patients with presence of DM and overweight status also displayed higher risk than non-deficient patients for risk of renal event.

**Conclusion:**

Vitamin D deficiency is associated with significantly increased risk of severe CKD stage and renal event.

## Introduction

Chronic kidney disease (CKD) is defined as the existence of structural or functional abnormalities of the kidney, with or without decreased estimated glomerular filtration rate (eGFR < 60 mL/min/1.73m^2^), lasting over three months (1); it is considered a significant worldwide health problem (2). The estimated prevalence of CKD in Korean adults is 8.2% according to the Korean National Health and Nutritional Examination Surveys (KNHANES) (3). In addition, Vitamin D deficiency is high among Korean adults (4), both physicians and patients are questioning whether supplement of vitamin D are needed.

Vitamin D, a steroid hormone known for its importance in bone metabolism, can be supplemented with diet, supplement, or produced in the body by dermal synthesis with UV from the precursor (5). Vitamin D deficiency has been related to numerous conditions such as fracture risk, diabetes, fractures, renal disease, cardiovascular disease, auto-immune disease, depression, and cancer (6, 7).

The causal relationship between vitamin D deficiency and decreased kidney function remains debates. Decreased kidney function is related to lower enzyme 1a-hydroxylase activity in the proximal tubule, which leads to decreased activation of 25(OH)D to 1,25(OH)_2_D (8). However, increasing evidence from scientific approaches has shown an association between low vitamin D levels and decreased estimated glomerular filtration (eGFR) (9, 10). Vitamin D is known to play an important role in maintaining homeostasis, which is associated with the renin-angiotensin system (RAS) (11). Several studies have shown that activation of the RAS system due to low vitamin D level leads to kidney disease, hypertension, cardiovascular disease, insulin resistance, and increased mortality (12, 13). In fact, 2017 Kidney Disease Improving Global Outcomes (KDIGO) experts carefully suggested (level of evidence 2C) checking and supplementing low vitamin D levels in CKD and dialysis patients (14).

Some observational studies and guidelines have suggested a possible association between low vitamin D level and CKD (15–17). However, in most studies and guidelines, the causality between low vitamin D levels and risk of renal event could not be explained because this relationship was analyzed through a cross-sectional study or focused on dialysis patients, or did not distinguish the stage of CKD (early versus advanced stage) (15–17).

The purpose of this study was twofold. First, to evaluate the relationship between patients with 25(OH)D deficiency and CKD stages at baseline. Second, to analyze longitudinal follow-up data to elucidate the association between patients with 25(OH)D deficiency and risk of renal event, which is defined as a 50% decline in eGFR from the baseline value or the onset of CKD stage 5 across the follow-up. Further, controlling the effects of confounding on vitamin D levels using propensity score matching analysis (PSM), we evaluated the relationship between patients with 25(OH)D deficiency and severe CKD stage and renal event. We also investigated the associations of patients with 25(OH)D deficiency and risk of renal event according to diabetes and overweight status.

## Materials and methods

### Study design and subjects

We collected baseline data for 2,238 non-dialysis dependent patients with CKD from stage G1 to 5, who were enrolled in a prospective cohort study [KoreaN Cohort Study for Outcome in Patients With Chronic Kidney Disease] from 2011 to 2015 in collaboration with a multicenter, patient-based, prospective-cohort study. The detailed design and methods were previously published (18). Among 2,238 (KNOW-CKD) cohort study patients, a total of 2,144 patients with serum 25(OH)D was included in this study (Supplementary Figure 1). This study was conducted according to guidelines established by the Declaration of Helsinki. All patients gave written informed consent for inclusion before they participated in the study. The study protocol was approved by the institutional review board of each participating clinical center: Seoul National University Hospital (1104-089-359), Seoul National University Bundang Hospital (B-1106/129–008), Kangbuk Samsung Medical Center (2011–01-076), Yonsei University Severance Hospital (4-2011-0163), Seoul St. Mary’s Hospital (KC11OIMI0441), Eulji General Hospital (201105-01), Gil Hospital (GIRBA2553), Pusan Paik Hospital (11-091), Chonnam National University Hospital (CNUH-2011-092) in 2011.

### Data collection

Data regarding personal and family history, anthropometric measurements, cardiac evaluation, radiological imaging, and baseline laboratory results were extracted from the electronic data management system (PhactaX: http://www.phactaX.org) with assistance from the Division of data management at Seoul National University Medical Research Collaborating Center. Serum samples were harvested and sent immediately to the central laboratory of Lab Genomics, Seongnam, Republic of Korea. Urine samples were also immediately sent to the central laboratory for KNOW-CKD. The reliability of biomarker analysis was previously reported (18).

### Main exposure variables and other biomarker

Serum 25(OH)D level was measured by electrochemiluminescence immunoassay (ECLIA), using an ADVIA Centaur Vitamin D Total assay reagents (Siemens, NY, USA). 25(OH)D deficiency was defined as level < 15 ng/mL, as considered in the National Kidney Foundation’s Kidney Disease Outcomes Quality Initiative (KDOQI) guidelines (19). Non-deficient levels of 25(OH)D was ≥ 15 ng/mL. Limit of detection for 25(OH)D level was 3.20 ng/mL (18).

Serum c-terminal FGF23 level was measured using enzyme-linked immunosorbent assay (ELISA; Immunotopics, San Clemente, CA, USA). Serum levels of klotho were measured using an ELISA kit (Immuno-Biological Laboratories Co., Gunma, Japan). (18).

### Outcome variables

Serum creatinine was measured by an IDMS-traceable method at a central laboratory and eGFR was calculated using the Chronic Kidney Disease Epidemiology Collaboration formula (CKD-EPI) (20). Mild CKD stage (Stage 1/2) was defined as eGFR ≥ 60 mL/min/1.73 m^2^. Moderate CKD stage (Stage 3A/3B) was defined as 30 ≤ eGFR < 60 mL/min/1.73 m^2^ and severe CKD stage (Stage 4/5) was defined as eGFR of less than 30 mL/min/1.73 m^2^.

Renal event was a composite of the first occurrence of a 50% decline in eGFR from the baseline value or the onset of CKD stage 5 (initiation of dialysis or kidney transplantation) across the follow-up period.

### Statistical analysis

Characteristics of patients are described by binary of 25(OH)D, before and after propensity score matching analysis. Continuous variables are expressed as mean and standard deviation for t-tests, and categorical variables are reported as number of patients and percentage for the chi-squared test. To examine the association of 25(OH)D with CKD stages and risk of renal event, polytomous logistic regression and Cox proportional hazard models were constructed to calculate odds ratio (ORs) and hazard ratio (HRs), using non-deficiency vitamin D levels as the reference. Patients who were lost to follow-up were censored at the date of the last hospital examination. This study was adjusted for clinically important confounding factors: age, sex, baseline eGFR, serum phosphorus levels, serum intact parathyroid hormone (iPTH), serum fibroblast growth factor 23 (FGF23), klotho, random urine albumin-to-creatinine ratio (UACR), 24-h urine protein, diabetes mellitus (DM), cause of CKD, use of vitamin D supplements, and angiotensin converting enzyme inhibitors(ACEi)/angiotensin receptor blocker (ARBs) medications. Further, controlling the effects of confounding on vitamin D levels using propensity score matching analysis (PSM), we evaluated the relationship between vitamin D deficiency and severe CKD stage and risk of renal event. Confounding variables affecting age, sex, PTH, serum phosphorus, serum klotho, FGF23, and cause of CKD were matched in the two study groups using 1:1 propensity score matching analysis (PSM) and a caliper width of 0.01. We also examined multi-collinearity between independent variables with Pearson correlation coefficients and variance inflation factor. We also performed stratified analysis by DM (No vs. Yes), and overweight (BMI < 23 kg/m^2^ *vs.* BMI ≥ 23 kg/m^2^) status using Cox regression models. P-interaction term was calculated to determine interaction between DM, overweight and each vitamin D level. The spline model was adjusted for age, sex, and confounding factors. The survival probability was calculated with the Kaplan-Meier (KM) method. To analyze the association with vitamin D on the risk of renal events, a Cox regression model was constructed by controlling the following 8 risk factors, which were statistically significant in each univariable analysis: age, sex, baseline eGFR, cause of CKD, 24-h proteinuria, intact PTH and serum klotho. Each model was constructed by backward Cox regression model. In order to observe the effect of vitamin D on the risk of renal events under adjustment for 25(OH)D levels and other risk factors, we constructed a vitamin D-epidemiologic-clinical model which was a full multivariable model. In addition, we evaluated the discriminatory accuracy of the only vitamin D model, vitamin D-epidemiological model, and a vitamin D-epidemiologic-clinical model in predicting risk of renal events using Harrell’s C index.

P-value less than 0.01 were considered statistically significant. All statistical analyses were performed using SAS version 9.4 (SAS Institute Inc., Cary, NC, USA) and spline plots were drawn by R version 4.2.1 (http://www.r-project.org). *P*-values < 0.05 were considered statistically significant.

## Results

### Baseline characteristics of the study subjects

Baseline characteristics of the study population are described Supplementary Table 1. Compared with non-deficient vitamin D patients, patients with deficiency were more likely to be male; have higher SBP, DBP, UACR, UPCR, 24-h urine protein, serum creatinine, phosphorus, potassium, total cholesterol, i-PTH, FGF23, klotho, hepcidin, angiotensin levels; have lower eGFR, hemoglobin, uric acid, albumin, calcium and sodium levels. The PSM study showed that all variables were not statistically different according to vitamin D levels.

### Association between vitamin D deficiency and severe CKD stage

Table 1 presents the associations of patients with 25(OH)D deficiency with severe CKD stage. We found increased odds for severe CKD stage in patients with vitamin D deficiency levels compared to patients with non-deficient levels (OR = 1.30, 95% CI = 1.01-1.69). In addition, Table 1 shows the likelihood of having severe CKD stage relative to mild CKD stage by serum levels of vitamin D biomarkers using PSM. Patients with 25(OH)D deficiency levels were not statistically significant associated with increased odds of severe CKD stage compared to patients with non-deficient levels (OR = 1.44, 95% CI = 0.98-2.13).

**TABLE 1:** Association between serum vitamin D levels and moderate (Stage 3A/3B) and severe CKD (Stage 4/5) stage in the Korean Cohort Study for Outcome in Patients With Chronic Kidney Disease (KNOW-CKD) study, 2011-2015.

Vitamin D biomarkers	CKD Stage 1/2	CKD Stage 3A/3B		CKD Stage 4/5	
	**N (%)**	**N (%)**	**OR (95% CI) ^1^**	**N (%)**	**OR (95% CI) ^1^**
**Total cohort**					
**25(OH)D**					
≥ 15 *ng/mL*	474 (62.8)	500 (62.5)	1.00	320 (54.3)	1.00
< 15	281 (37.2)	300 (37.5)	1.06 (0.84-1.33)	269 (45.7)	1.30 (1.01-1.69)
3T (≥ 19.4 *ng/mL*)	244 (32.3)	286 (35.7)	1.00	184 (31.2)	1.00
2T (14.1 – 19.3)	266 (35.2)	264 (33.0)	0.98 (0.74-1.26)	188 (31.9)	1.04 (0.77-1.41)
1T (< 14.1)	245 (32.4)	250 (31.2)	0.95 (0.72-1.25)	217 (36.8)	1.15 (0.84-1.57)
**PS matching cohort ^2^**					
**25(OH)D**					
≥ 15 *ng/mL*	158 (55.8)	130 (49.4)	1.00	85 (42.5)	1.00
< 15	125 (44.2)	133 (50.6)	1.21 (0.86-1.71)	115 (57.5)	1.44 (0.98-2.13)

Chronic kidney disease (CKD); 25-hydroxyvitamin (25(OH)D); 1,25-dihydroxyvitamin (1,25(OH)_2_D); Propensity score (PS) matching; Systolic blood pressure (SBP); Fibroblast growth factor 23 (FGF23); Urine albumin creatinine ratio (UACR); Diabetes mellitus (DM); Angiotensin receptor blockers (ARB) medication.

^1^Logistic regression model for total cohort was adjusted for age, sex, systolic blood pressure, serum phosphorus, Fibroblast growth factor 23, klotho, cause of chronic kidney disease, UACR, Diabetes mellitus, use of vitamin supplements and ARB medications. Logistic regression model for PS matching cohort were adjusted for baseline eGFR, SBP, DM, 24 h-Urine protein, UACR, and use of ARB medications.

^2^For propensity score (PS) matching cohort, PS matching was performed using the variables such as low vitamin D and high vitamin D biomarker levels.

### Association between vitamin D deficiency and renal event

**FIGURE 1:**
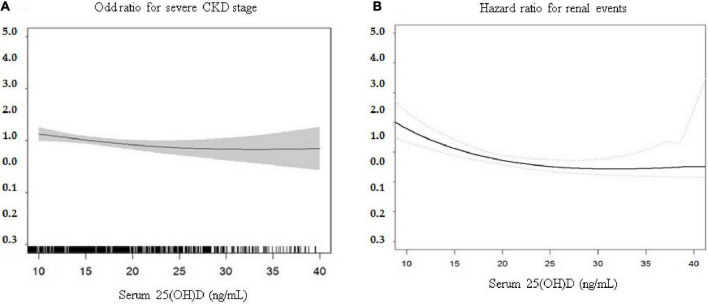
Multivariate association of continuously measured 25(OH)D levels (ng/mL) and **(A)** severe CKD stages and **(B)** risk of renal events. The relationship between serum vitamin D levels (continuous scale) and risk of renal event were non-linear after multivariate adjustment. CKD, Chronic kidney disease; 25(OH)D, 25-hydroxyvitamin D.

**FIGURE 2:**
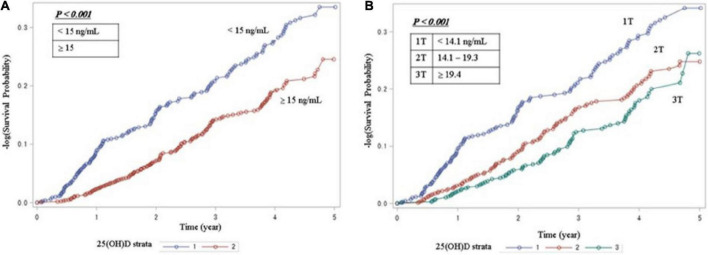
Kaplan-Meier survival analysis for serum vitamin D levels **(A)** Survival curve for renal event by binary of 25(OH)D. **(B)** Survival curve for renal event by tertiles of 25(OH)D. The Kaplan-Meier curves show that the patient with vitamin D deficiency group had a significantly higher cumulative incidence of renal events (*P* < 0.001). 25(OH)D, 25-hydroxyvitamin D.

Table 2 shows that 342 patients reached the composite outcome of renal event. Patients with 25(OH)D deficiency levels had a significantly increased risk of renal events compared to patients with non-deficient levels (HR = 1.64, 95% CI = 1.32-2.05). Table 2 also shows the risk of renal event according to serum levels of vitamin D biomarkers using PSM. After PSM, patients with 25(OH)D deficiency levels had no significant association with renal event (HR = 1.52, 95% CI = 0.88-2.65). The relationship between serum vitamin D levels (continuous scale) and severe CKD stage and risk of renal event were non-linear after multivariate adjustment (Figure 1). The Kaplan-Meier survival curves show statistically significant difference in renal event probability among the binary and tertiles of serum 25(OH)D levels (p < 0.001) (Figure 2). Thus, this model has a certain practicability and reliability in evaluating prognosis.

**TABLE 2:** Association between serum vitamin D levels and renal event in the Korean Cohort Study for Outcome in Patients With Chronic Kidney Disease (KNOW-CKD) study, 2011-2015.

Vitamin D biomarkers	CKD cohort		Renal event^1^	
	**N**	**PY**	**N**	**HR (95% CI)^2^**
**Total cohort**				
**25(OH)D**				
≥ 15 *ng/mL*	1,294	3,891	171	1.00
< 15	850	2,432	171	1.64 (1.32-2.05)
3T (≥ 19.4 *ng/mL*)	630	2,078	84	1.00
2T (14.1 – 19.3)	604	2,269	114	1.25 (0.94-1.66)
1T (< 14.1)	568	1,977	144	1.82 (1.38-2.41)
**PS matching cohort ^3^**				
**25(OH)D**				
≥ 15 *ng/mL*	373	607	19	1.00
< 15	373	621	42	1.52 (0.88-2.65)

Chronic kidney disease (CKD); 25-hydroxyvitamin (25(OH)D); 1,25-dihydroxyvitamin (1,25(OH)_2_D); Propensity score (PS) matching; Systolic blood pressure (SBP); Fibroblast growth factor 23 (FGF23); Urine albumin creatinine ratio (UACR); Diabetes mellitus (DM); Angiotensin receptor blockers (ARB) medication.

^1^Renal events were a composite of the first occurrence of a 50% decline in eGFR from the baseline value or the onset of ESRD (the initiation of dialysis or kidney transplantation) during follow-up period.

^2^Cox proportional hazards model were adjusted for age, sex, baseline eGFR, SBP, serum phosphorus, FGF23, klotho, cause of CKD, 24h-Urine protein, UACR, Diabetes mellitus, use of vitamin supplements and ARB medications. Cox proportional hazards model for PS matching cohort were adjusted for baseline eGFR, SBP, DM, 24 h-Urine protein, UACR, and use of ARB medications.

^3^For propensity score (PS) matching cohort, PS matching was performed using the variables such as low vitamin D and high vitamin D biomarker levels.

### Association between vitamin D deficiency and severe CKD stage according to diabetes and overweight

Compared to the non-DM, the diabetic CKD showed stronger associations between CKD stage 4 and 5 and patients with 25(OH) deficiency (OR = 1.59, 95% CI = 1.13-2.25, p-interaction = 0.02).

In this study, compared to the non-overweight (BMI < 23), the patients with overweight (BMI ≥ 23) status demonstrated stronger associations between CKD stage 4 and 5 and patients with 25(OH) deficiency (OR = 1.41, 95% CI = 1.03-1.92, p-interaction = 0.30). On the other hand, none of the patients with 25(OH)D deficiency showed a significant association with the risk of CKD stage 3A and 3B according to DM and overweight status (Table 3).

**TABLE 3:** Association between serum vitamin D levels and moderate (Stage 3A/3B) and severe CKD (Stage 4/5) stage according to diabetes and overweight status in the Korean Cohort Study for Outcome in Patients With Chronic Kidney Disease (KNOW-CKD) study, 2011-2015.

Vitamin D biomarkers	CKD Stage 1/2	CKD Stage 3A/3B		CKD Stage 4/5		CKD Stage ½	CKD Stage 3A/3B		CKD Stage 4/5		P-interaction^2^
	**N (%)**	**N (%)**	**OR (95% CI) ^1^**	**N (%)**	**OR (95% CI) ^1^**	**N (%)**	**N (%)**	**OR (95% CI) ^1^**	**N (%)**	**OR (95% CI) ^1^**	
	**DM**					**Non-DM**					
**25(OH)D**											
≥ 15 *ng/mL*	243 (64.5)	303 (60.9)	1.00	197 (50.7)	1.00	231 (61.1)	197 (65.0)	1.00	123 (61.2)	1.00	
< 15	134 (35.3)	194 (39.0)	1.20 (0.88-1.64)	191 (49.2)	1.59 (1.13-2.25)	147 (38.9)	106 (35.0)	0.91 (0.64-1.30)	78 (38.8)	0.85 (0.55-1.30)	0.02
3T (≥ 19.4 *ng/mL*)	135 (35.8)	178 (35.8)	1.00	111 (28.6)	1.00	109 (28.8)	108 (35.6)	1.00	73 (36.3)	1.00	
2T (14.1 – 19.3)	118 (31.3)	152 (30.6)	0.99 (0.69-1.43)	118 (30.4)	1.21 (0.80-1.84)	148 (39.1)	112 (36.9)	0.88 (0.59-1.32)	70 (34.8)	0.75 (0.46-1.21)	
1T (< 14.1)	124 (32.9)	167 (33.6)	1.06 (0.73-1.53)	159 (40.9)	1.39 (0.92-2.10)	121 (32.0)	83 (27.4)	0.75 (0.48-1.16)	58 (38.9)	0.59 (0.35-1.01)	
	**BMI ≥ 23 kg/m^2^**					**BMI < 23 kg/m^2^**					
**25(OH)D**											
≥ 15 *ng/mL*	311 (63.7)	369 (64.2)	1.00	201 (51.9)	1.00	160 (61.3)	127 (58.3)	1.00	118 (58.7)	1.00	
< 15	177 (36.3)	206 (35.8)	0.97 (0.73-1.28)	186 (48.1)	1.41 (1.03-1.92)	101 (38.7)	91 (41.7)	1.38 (0.89-2.13)	83 (41.3)	1.29 (0.81-2.05)	0.30
3T (≥ 19.4 *ng/mL*)	160 (32.8)	199 (34.6)	1.00	119 (30.7)	1.00	83 (31.8)	84 (38.5)	1.00	64 (31.8)	1.00	
2T (14.1 – 19.3)	175 (35.9)	201 (35.0)	1.00 (0.73-1.37)	115 (29.7)	0.95 (0.65-1.38)	89 (34.1)	62 (28.4)	0.90 (0.54-1.50)	73 (36.3)	1.39 (0.80-2.39)	
1T (< 14.1)	153 (31.4)	175 (30.4)	0.92 (0.66-1.29)	153 (39.5)	1.14 (0.78-1.67)	89 (34.1)	72 (33.0)	0.97 (0.58-1.62)	64 (31.8)	1.13 (0.65-1.99)	

Chronic kidney disease (CKD); 25-hydroxyvitamin (25(OH)D); Systolic blood pressure (SBP); Fibroblast growth factor 23 (FGF23); Urine albumin creatinine ratio (UACR); Diabetes mellitus (DM); Angiotensin receptor blockers (ARB) medication.

^1^Logistic regression model for total cohort was adjusted for age, sex, systolic blood pressure, serum phosphorus, Fibroblast growth factor 23, klotho, cause of chronic kidney disease, UACR, use of vitamin supplements and ARB medications.

^2^P-interaction term was calculated to determine interaction between DM, overweight and each vitamin D level.

### Association between vitamin D deficiency and renal event according to diabetes and overweight

As shown in Table 4, patients with DM, and overweight (BMI ≥ 23) status appeared to have a greater risk for renal event in the context of vitamin D deficiency. The associations of 25(OH)D deficiency with risk of renal event were stronger in patients with DM (HR = 1.81, 95% CI = 1.38-2.38, p < 0.01 for interaction between 25(OH)D and DM for renal event; and overweight status (HR = 1.89, 95% CI = 1.44-2.48, p < 0.01 for interaction between 25(OH)D and overweight for renal event.

**TABLE 4:** Association between serum vitamin D levels and renal event according to diabetes and overweight status in the Korean Cohort Study for Outcome in Patients With Chronic Kidney Disease (KNOW-CKD) study, 2011-2015.

Vitamin D Biomarkers	Renal event ^1^		Renal event ^1^		
	**N**	**HR (95% CI)**	**N**	**HR (95% CI)**	**P-interaction ^4^**
**Stratification by DM^2^**	**DM**		**Non-DM**		
**25(OH)D**					
≥ 15 *ng/mL*	101	1.00	70	1.00	
< 15	128	1.81 (1.38-2.38)	43	1.12 (0.75-1.67)	< 0.01
**Stratification by BMI^3^**	**BMI ≥ 23 kg/m^2^**		**BMI < 23 kg/m^2^**		
**25(OH)D**					
≥ 15 *ng/mL*	105	1.00	66	1.00	
< 15	120	1.89 (1.44-2.48)	51	1.08 (0.73-1.60)	< 0.01

Chronic kidney disease (CKD); 25-hydroxyvitamin (25(OH)D); Propensity score (PS) matching; Systolic blood pressure (SBP); Fibroblast growth factor 23 (FGF23); Urine albumin creatinine ratio (UACR); Diabetes mellitus (DM); Body mass index (BMI); Angiotensin receptor blockers (ARB) medication.

^1^Renal events were a composite of the first occurrence of a 50% decline in eGFR from the baseline value or the onset of ESRD (the initiation of dialysis or kidney transplantation) during follow-up period.

^2^Adjusted for age, sex, baseline GFR, SBP, serum phosphorus, FGF23, klotho, cause of CKD, 24h-Urine protein, UACR, use of vitamin supplements and ARB medications.

^3^Adjusted for age, sex, baseline GFR, SBP, serum phosphorus, FGF23, klotho, cause of CKD, 24h-Urine protein, UACR, Diabetes mellitus, use of vitamin supplements and ARB medications.

^4^P-interaction term was calculated to determine interaction between DM, overweight and each vitamin D level.

### Discriminatory accuracy of models on predicting the risk of renal events

**FIGURE 3:**
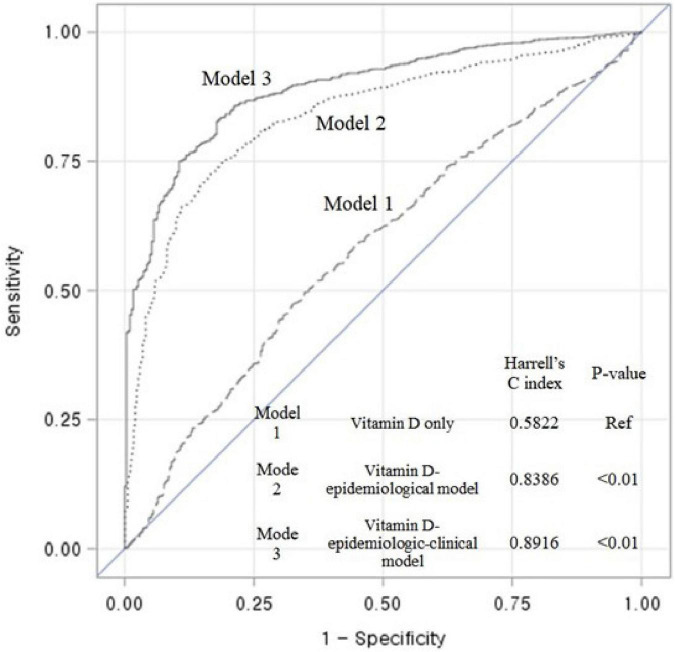
Receiver operating characteristic (ROC) curves and Harrell’s C-index showing the discriminant accuracy of each model for the ability to distinguish renal events in entire cohort; Model 1 (vitamin D model): Function**(Y)** = β1[25(OH)D], Function(Y) = Log ((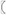 Hazard 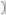 _Exposed)/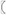 Hazard 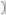 _(Non-Exposed)); Model 2 (Vitamin D-epidemiological model): Function(Y) = β1[25(OH)D] + β2[Age] + β3[Sex] + β4[Baseline eGFR]; Model 3 (Vitamin D-epidemiologic -clinical model): Function(Y) = β1[25(OH)D] + β2[Age] + β3[Sex] + β4[Baseline eGFR] + β5[Cause of CKD] + β6[24-h proteinuria] + β7[intact PTH] + β8 [Klotho]; A full multivariable model shows the highest differential accuracy among the three models predicting the risk of renal events. The C-index of the model was significantly different from that of the vitamin D only model (*p*-value < 0.01). 25(OH)D, 25-hydroxyvitamin D; eGFR, estimated glomerular filtration.

With the ability to discriminate renal events for the epidemiological model, a multivariate model controlled by 25(OH)D levels and other risk factors, the Harrell’s C-index at long term follow up was as follow: Model 1 (vitamin D only) was constructed with 1 variable and the Harrell’s C-index was 0.5822. Model 2 (vitamin D-epidemiological model) was constructed with 4 variables and the Harrell’s C-index was 0.8386. Model 3 (vitamin D-epidemiologic-clinical model) was constructed with 8 variables and the Harrell’s C-index was 0.8916. A full multivariable model shows the highest differential accuracy among the three models predicting the risk of renal events. The C-index of the model was significantly different from that of the vitamin D only model (p-value < 0.01). (Figure 3).

## Discussions

In this study, we found that patients with 25(OH)D deficiency levels was significantly associated with severe CKD stage and increased risk of renal event. Furthermore, vitamin D deficiency patients with presence of DM and overweight status (≥ 23 kg/m^2^) also displayed higher risk than non-deficient patients for severe CKD stage and risk of renal event. None of the patients with 25(OH)D deficiency showed a significant association with the risk of CKD stage 3A and 3B.

Our findings are consistent with a number of previous studies. A cross-sectional studies conducted in France and Thailand showed that 25(OH)D deficiency was related to decreased GFR and developing ESRD (15, 16). A cohort study in the United States found that low 25(OH)D level was associated with development of ESRD (21). Similarly, another cohort study in Italy reported that low 25(OH)D level is an independent predictor of CKD progression (22).

On the other hand, previous studies found no association between low 25(OH)D level and CKD progression. A longitudinal patient cohort study reported that African Americans with low 25(OH)D levels were not associated with ESRD or doubling of serum creatinine (23). The Framingham Offspring cohort study showed similar results (24). The biggest difference between the former studies (15, 16, 21, 22) including our study and the latter studies (23, 24), is that the former studies (15, 16, 21, 22) reported that 25(OH)D deficiency is associated with risk of renal event while the latter studies (23, 24) showed that 25(OH)D deficiency is not associated with the risk of ESRD in the general population, where incidence of ESRD is less common. These inconsistencies in results can be attributed to various reasons, including differences in the general characteristics of the study participants, outcome variables, and confounding variables.

In addition, we found interactions between vitamin D deficiency and DM or between vitamin D deficiency and overweight for the risk of renal event. DM is one of the most common causes of CKD. Low vitamin D levels are related to insulin resistance and impaired pancreatic islet B-cell function (25). The effect is on upregulation of the insulin receptor gene and calcium and phosphorus metabolism (26). Also, an adequate vitamin D levels suppresses RAS system by reducing the expression AT1 receptors and by inhibiting renin synthesis in the kidney. It could lead to nephron-protective effects by reducing proteinuria and decreasing CKD progression (27). Previous studies reported that 25(OH)D deficiency is associated with type 2 DM (28). Since DM can induce increased risk of renal event, patients with DM may be more susceptible to vitamin D deficiency with regards to CKD than those without DM. Also, high BMI is one of the strongest risk factors for CKD (29). Previous study has shown that adipose tissue may increase the secretion of inflammatory cytokines or chemokine (MCP-1, IL-6, IL-1beta, TNF-a) and its plausible influence on the course of CKD (30). Overweight or obesity status is directly related to hypertension and diabetes status, the metabolic disorders responsible for the ESRD (31). One animal study presents that association between vitamin D deficiency and obesity impairs the renal function, hemodynamics, and metabolic parameters in the High-fat vitamin D deficient (H + VDD) rats. Obesity associated to vitamin D deficiency aggravated the renal inflammation associated to renal progression (32).

While the exact mechanism for vitamin D deficiency and risk of renal event is unclear, several biological mechanisms have been proposed. Vitamin D is converted enzymatically in the liver to 25(OH)D, the major circulating form, and then in the kidney to 1,25(OH)_2_D, the active form of vitamin D (33). Low levels of 1,25(OH)_2_D levels in the body can lead to deterioration of the RAS system by inducing abnormal metabolic profiles such as increasing renin, proteinuria, blood pressure, insulin resistance, and renal injury (34–36). For example, vitamin D supplementation decreases renin receptor and renin expression in rat models of CKD (37). Another possible mechanism is that low levels of 25(OH)D may modulate inflammation and oxidative stress and reduce fibroblast activation (13). In addition, CKD progression has been related to lower 25(OH)D reuptake and reduction of intracrine 1,25(OH)_2_D levels in the renal proximal tubules (38). Also, 1,25(OH)_2_D levels are known to reduce expression of the nuclear factor kB and promote a shift in T-helper cell response from T-helper 1 cell to T-helper 2 cell (39). Therefore, this reduces T-helper 1 cell-mediated tissue damage and increases production of T-helper 2 cell immunomodulatory cytokines (40). A genetic predisposition that affects higher vitamin D binding protein (DBP) was suggested as a plausible mechanism in the association between vitamin D level and risk of renal events (41). DBP is facilitated by receptor-mediated endocytosis. In the renal proximal tubule, cublin and megalin induce uptake of extracellular ligands. Deficiency of these proteins results in increased vitamin D excretion in the urine (42). More studies are needed to understand these effects in humans.

Several limitations should be considered when interpreting our results. First, serum vitamin D levels are generally affected by seasonal variation and the extent of UV exposure should be considered (43). However, our study could not be adjusted for seasonal variation. Second, we did not adjust our data for patient nutritional status. Nutritional status may also contribute to vitamin D status in CKD patients. Malnutrition is a typical finding in patients with CKD. Uremia may be related to impaired gastrointestinal absorption of vitamin D (44). Third, we conducted a single measurement of vitamin D levels at baseline. Our data in the present study did not have enough repeated measures to perform the analysis. Fifth, this study is an observational study. Therefore, it is possible that potential confounding factors were not correctly adjusted. To reduce this possibility, we conducted PSM analysis and found consistent results. Finally, the KNOW-CKD cohort consists only of Korean CKD patients and our findings may not be generalizable to other ethnic groups.

Despite these limitations, the present study has several strengths. First, our study used a well-designed patient-based prospective cohort (the KNOW-CKD) representative of Korean CKD patients. In addition, serum vitamin D levels can be easily obtained in the clinical setting and treatment of vitamin D deficiency/insufficiency is simple and inexpensive. This study should be taken into consideration by researchers and clinicians in order to improve CKD patient outcomes.

## Conclusion

The present study suggests that vitamin D deficiency is significantly associated with risk of severe CKD stage and renal event.

## Data availability statement

The raw data supporting the conclusions of this article will be made available by the authors, without undue reservation.

## Ethics statement

The studies involving human participants were reviewed and approved by Seoul National University Hospital (1104-089-359), Seoul National University Bundang Hospital (B-1106/129–008), Kangbuk Samsung Medical Center (2011–01-076), Yonsei University Severance Hospital (4-2011-0163), Seoul St. Mary’s Hospital (KC11OIMI0441), Eulji General Hospital (201105-01), Gil Hospital (GIRBA2553), Pusan Paik Hospital (11-091), Chonnam National University Hospital (CNUH-2011-092). The patients/participants provided their written informed consent to participate in this study.

## Author contributions

JL, EB, SK, K-HO, and SP designed the study. JL, K-HO, and SP conducted the data analysis and drafted the manuscript and wrote the manuscript. K-HO and SP provided the supervision and mentorship. All authors supported the interpretation of results, provided important intellectual content and revised the final version of the manuscript and also provided final approval of the version to be published.
