# Supplementary figures and images for: The association between vitamin D deficiency and risk of renal event: Results from the Korean cohort study for outcomes in patients with chronic kidney disease (KNOW-CKD)
Source: Front Med (Lausanne). 2023 Feb 16;10:1017459. doi: 10.3389/fmed.2023.1017459 (PMC9978501; doi:10.3389/fmed.2023.1017459)

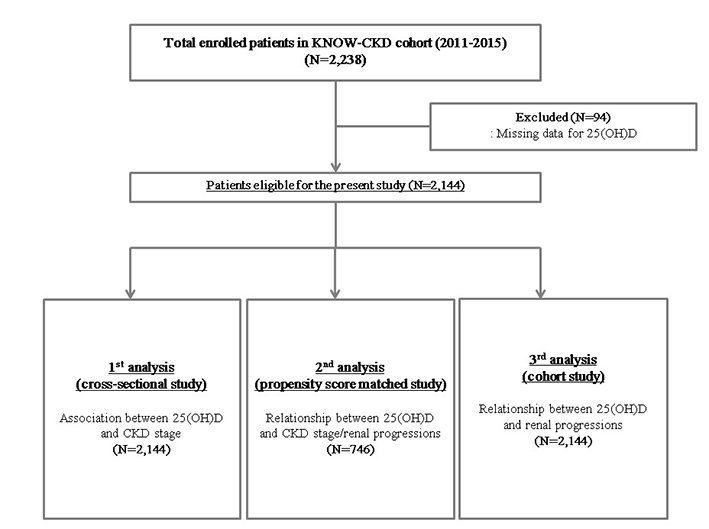

Supplement: Supplementary file 1 [file Image_1.JPEG]
