# Supplementary material for: The association between vitamin D deficiency and risk of renal event: Results from the Korean cohort study for outcomes in patients with chronic kidney disease (KNOW-CKD)
Source: Front Med (Lausanne). 2023 Feb 16;10:1017459. doi: 10.3389/fmed.2023.1017459 (PMC9978501; doi:10.3389/fmed.2023.1017459)
Supplement: Supplementary file 2 [file Table_1.DOCX]

**Supplementary Table 1.** Baseline characteristics of serum levels of vitamin D biomarkers before and after propensity score matching analysis in the Korean Cohort Study for Outcome in Patients With Chronic Kidney Disease (KNOW-CKD) study, 2011-2015.

|  | **Total CKD cohort** | | | **Matched sets by PSM** | | | | | |
| --- | --- | --- | --- | --- | --- | --- | --- | --- | --- |
|  | **25(OH)D ng/mL** | | | |  | **25(OH)D ng/mL** | | |  |
|  | **< 15** | **15 ≤** | P^1^ | |  | **< 15** | **15 ≤** | P^1^ |  |
|  | N=850 | N=1294 |  | |  | N=372 | N=372 |  |  |
|  |  |  |  | |  |  |  |  |  |
|  | Mean (SD) | Mean (SD) |  | |  | Mean (SD) | Mean (SD) |  |  |
| Age (years) | 52.81 (12.70) | 54.14 (11.90) | 0.01 | |  | 53.86 (12.77) | 53.89 (11.96) | 0.97 |  |
| eGFR (ml/min/1.73m^2^) | 51.90 (32.87) | 54.02 (29.26) | 0.01 | |  | 51.82 (31.81) | 52.99 (31.02) | 0.62 |  |
| Body mass index (kg/m^2^) | 24.62 (3.66) | 25.42 (3.20) | 0.50 | |  | 24.94 (3.68) | 24.71 (3.38) | 0.37 |  |
| SBP (mmHg) | 130.6 (17.56) | 127.3 (15.49) | <0.01 | |  | 128.60 (15.67) | 127.10 (15.89) | 0.19 |  |
| DBP (mmHg) | 77.49 (11.56) | 76.76 (10.81) | 0.03 | |  | 76.94 (10.70) | 78.23 (10.89) | 0.10 |  |
| UACR | 900.6 (1427.4) | 599.3 (1163.2) | <0.01 | |  | 676.9 (273.7) | 593.0 (278.0) | 0.53 |  |
| UPCR | 1.77 (2.29) | 1.07 (1.73) | <0.01 | |  | 1.15 (2.93) | 1.07 (1.82) | 0.05 |  |
| 24-h urine protein (mg/day) | 1830.8 (2698.0) | 1051.8 (1618.6) | <0.01 | |  | 1538.8 (1897.2) | 1231.5 (1886.9) | 0.05 |  |
| Serum creatinine (mg/dL) | 2.00 (1.37) | 1.81 (1.00) | <0.01 | |  | 1.98 (1.23) | 1.82 (0.95) | 0.05 |  |
| Serum hemoglobin (g/dL) | 12.46 (2.02) | 13.07 (1.98) | <0.01 | |  | 12.47 (2.11) | 12.96 (2.06) | 0.64 |  |
| Serum uric acid (mg/dL) | 6.93 (1.98) | 7.09 (1.88) | 0.06 | |  | 6.77 (1.90) | 6.84 (1.91) | 0.59 |  |
| Serum albumin (g/dL) | 4.09 (0.49) | 4.19 (0.36) | <0.01 | |  | 4.06 (0.55) | 4.10 (0.39) | 0.06 |  |
| Serum calcium (pg/mL) | 9.01 (0.60) | 9.18 (0.47) | <0.01 | |  | 9.02 (0.62) | 9.10 (0.48) | 0.66 |  |
| Serum phosphorus (g/dL) | 3.83 (0.71) | 3.58 (0.62) | <0.01 | |  | 3.84 (0.75) | 3.72 (0.63) | 0.72 |  |
| Serum sodium (mEq/L) | 140.7 (2.69) | 140.9 (2.21) | 0.01 | |  | 140.5 (2.49) | 140.7 (2.23) | 0.85 |  |
| Serum potassium (mEq/L) | 4.66 (0.62) | 4.58 (0.54) | <0.01 | |  | 4.69 (0.61) | 4.62 (0.54) | 0.12 |  |
| Total cholesterol (mg/dL) | 179.0 (43.97) | 171.0 (35.56) | <0.01 | |  | 180.0 (46.88) | 176.97 (37.48) | 0.41 |  |
| HDL cholesterol (mg/dL) | 49.55 (17.32) | 49.07 (14.13) | 0.47 | |  | 49.02 (17.01) | 48.82 (13.32) | 0.86 |  |
| Intact PTH (pg/mL) | 90.38 (102.6) | 57.36 (40.77) | <0.01 | |  | 80.84 (103.70) | 68.62 (42.62) | 0.07 |  |
| FGF23 (RU/mL) | 38.92 (51.11) | 20.20 (32.30) | <0.01 | |  | 32.85 (32.72) | 23.04 (23.67) | 0.07 |  |
| Klotho (pg/mL) | 562.4 (368.3) | 543.0 (367.6) | <0.01 | |  | 519.3 (205.8) | 505.5 (195.6) | 0.67 |  |
| Hepcidin (ng/mL) | 21.78 (21.60) | 18.70 (18.97) | <0.01 | |  | 19.85 (19.24) | 16.49 (16.62) | 0.26 |  |
| Angiotensin (ug/mL) | 30.58 (71.67) | 22.86 (54.66) | 0.02 | |  | 33.56 (91.74) | 21.35 (44.20) | 0.31 |  |
| Bone mineral density |  |  |  | |  |  |  |  |  |
| Total Hip T-score | 0.07 (1.32) | 0.24 (1.23) | <0.01 | |  | 0.14 (1.35) | 0.23 (1.28) | 0.38 |  |
| L1-L4 T-score | -0.06 (1.53) | 0.02 (1.48) | 0.31 | |  | 0.06 (1.63) | 0.02 (1.51) | 0.18 |  |
| Femur neck T-score | -0.36 (1.33) | -0.25 (1.25) | 0.05 | |  | -0.31 (1.32) | -0.32 (1.21) | 0.13 |  |
|  | N (%) | N (%) |  | |  | N (%) | N (%) |  |  |
| Male | 201 (53.9) | 380 (66.7) | <0.01 | |  | 201 (53.9) | 209 (56.0) | 0.55 |  |
| eGFR (ml/min/1.73m^2^) |  |  |  | |  |  |  |  |  |
| CKD stage 1 | 58 (15.5) | 105 (18.4) | <0.01 | |  | 58 (15.5) | 73 (19.6) | 0.10 |  |
| CKD stage 2 | 67 (18.0) | 135 (23.7) |  | |  | 67 (18.0) | 85 (22.8) |  |  |
| CKD stage 3A | 61 (16.3) | 101 (17.7) |  | |  | 61 (16.3) | 58 (15.5) |  |  |
| CKD stage 3B | 72 (19.3) | 114 (20.0) |  | |  | 72 (19.3) | 72 (19.3) |  |  |
| CKD stage 4 | 90 (24.1) | 100 (17.5) |  | |  | 90 (24.1) | 71 (19.0) |  |  |
| CKD stage 5 | 25 (6.7) | 15 (2.6) |  | |  | 25 (6.7) | 14 (3.7) |  |  |
| Cause of CKD |  |  |  | |  |  |  |  |  |
| DM | 126 (33.8) | 116 (20.3) | <0.01 | |  | 126 (33.8) | 93 (24.9) | 0.08 |  |
| HTN | 59 (15.8) | 94 (16.5) |  | |  | 59 (15.8) | 74 (19.8) |  |  |
| GN | 122 (32.7) | 222 (38.9) |  | |  | 122 (32.7) | 129 (34.6) |  |  |
| PKD | 33 (8.8) | 98 (17.2) |  | |  | 33 (8.8) | 54 (14.4) |  |  |
| Unspecified | 33 (8.8) | 40 (7.0) |  | |  | 33 (8.8) | 23 (6.2) |  |  |
| HTN ^2^ | 3356 (95.4) | 538 (94.4) | 0.47 | |  | 356 (95.4) | 349 (93.6) | 0.26 |  |
| DM ^3^ | 251 (67.3) | 355 (62.3) | 0.12 | |  | 251 (67.3) | 239 (64.1) | 0.35 |  |
| Income ($) |  |  |  | |  |  |  |  |  |
| <1,500 | 87 (23.8) | 117 (21.0) | 0.58 | |  | 87 (23.8) | 80 (22.1) | 0.78 |  |
| 1,500 – 4,499 | 198 (54.1) | 306 (55.0) |  | |  | 198 (54.1) | 205 (56.6) |  |  |
| 5,000+ | 81 (22.1) | 133 (23.9) |  | |  | 81 (22.1) | 77 (21.3) |  |  |
| Vitamin D or calcium supplements | 11 (3.0) | 36 (6.3) | 0.02 | |  | 11 (3.0) | 26 (7.0) | 0.05 |  |
| ACEi use | 29 (7.8) | 67 (11.8) | 0.05 | |  | 29 (7.8) | 40 (10.7) | 0.16 |  |
| ARB use | 308 (82.8) | 451 (79.3) | 0.18 | |  | 308 (82.8) | 287 (77.2) | 0.05 |  |

Abbreviation: Chronic kidney disease (CKD); 25-hydroxyvitamin (25(OH)D); Propensity score (PS) matching; Body mass index (BMI); Systolic blood pressure (SBP); Diastolic blood pressure (SBP); Mean arterial pressure (MAP); Intact parathyroid hormone (iPTH); Bone mineral density (BMD); Fibroblast growth factor 23 (FGF23); Urine albumin creatinine ratio (UACR); Urine protein creatinine ratio (UPCR); Estimated glomerular filtration rate (eGFR); Diabetes mellitus (DM); Hypertension (HTN); Glomerulonephritis (GN); Polycystic Kidney (PKD); Angiotensin Converting Enzyme Inhibitors (ACEi); Angiotensin receptor blockers (ARB) medication;

1. For continuous variable, T-test was used. For categorical variables, chi-square test was used.
2. HTN defined as history of hypertension, SBP > 130 mmHg or DBP > 85 mmHg or taking anti-hypertensive medications.
3. DM defined as history of diabetes mellitus or elevated plasma glucose ≥ 100 mg/dl or taking anti-diabetic medications.
4. Propensity score (PS) matching by low vitamin D and high vitamin D biomarker levels: adjusted for baseline eGFR, SBP, DM, 24h-Urine protein, UACR, and use of ARB medications.

**Supplementary Table 2.** Vitamin D and renal events in the full multivariable model and additional risk factors

|  | **Model 1** | *p*-Value | **Model 2** | *p*-Value | **Model 3** | *p*-Value |
| --- | --- | --- | --- | --- | --- | --- |
| **25(OH)D** |  |  |  |  |  |  |
| ≥ 15 ng/mL | 1.00 |  | 1.00 |  | 1.00 |  |
| < 15 | 1.07 (1.00-1.17) | 0.03 | 1.05 (1.00-1.15) | 0.04 | 1.03 (1.00-1.13) | 0.04 |
| eGFR < 60 vs. 60+, baseline |  |  | 0.79 (0.74-0.84) | <0.01 | 0.80 (0.75-0.87) | <0.01 |
| Age |  |  | 1.00 (1.00-1.00) | 0.04 | 1.00 (1.00-1.00) | 0.02 |
| Sex (male) |  |  | 1.01 (0.93-1.11) | 0.07 | 1.01 (0.92-1.12) | 0.07 |
|  |  |  |  |  |  |  |
| Cause of CKD |  |  |  |  |  |  |
| Glomerulonephritis |  |  |  |  | 1.00 |  |
| Diabetic nephropathy |  |  |  |  | 1.09 (0.94-1.27) | 0.23 |
| Hypertensive nephropathy |  |  |  |  | 1.07 (0.93-1.22) | 0.36 |
| Polycystic kidney disease |  |  |  |  | 0.96 (0.81-1.13) | 0.61 |
| 24-h urine protein |  |  |  |  | 1.04 (0.84-1.28) | 0.73 |
| iPTH |  |  |  |  | 1.00 (1.00-1.00) | 0.04 |
| Klotho |  |  |  |  | 1.00 (1.00-1.00) | 0.01 |

1. Renal events were a composite of the first occurrence of a 50% decline in eGFR from the baseline value or the onset of ESRD (the initiation of dialysis or kidney transplantation) during follow-up period.
2. Model 1 (vitamin D model): Function(Y) = β_1_[25(OH)D], Function(Y) = Log ($\frac{\mathrm{Hazard}_{\mathrm{Exposed}}}{\mathrm{Hazard}_{Non-Exposed}}$); Model 2 (Vitamin D-epidemiological model): Function(Y) = β_1_[25(OH)D] + β_2_[Age] + β_3_[Sex] + β_4_[Baseline eGFR]; Model 3 (Vitamin D-epidemiologic-clinical model): Function(Y) = β_1_[25(OH)D] + β_2_[Age] + β_3_[Sex] + β_4_[Baseline eGFR] + β_5_[Cause of CKD] + β_6_[24-h proteinuria] + β_7_[intact PTH] + β_8_[Klotho];
